# The hepatic protective effects of tacrolimus as a rinse solution in liver transplantation: A meta-analysis

**DOI:** 10.1097/MD.0000000000015809

**Published:** 2019-05-24

**Authors:** Tao Guo, Junhao Lei, Jiamin Gao, Zhen Li, Zhisu Liu

**Affiliations:** a Department of Hepatobiliary and Pancreatic Surgery, Department of General Surgery; b Department of Urology Surgery, Zhongnan Hospital of Wuhan University, Wuhan; c Department of Emergency, Huashan Hospital, Fudan University, Shanghai, P.R. China.

**Keywords:** liver transplantation, meta-analysis, rinse solution, tacrolimus

## Abstract

**Background:**

Tacrolimus was used as a rinse solution against ischaemia-reperfusion injury (IRI) in liver transplantation for years but its protective effects remain controversies.

**Methods:**

We conducted literature retrieval in electronic databases including MEDLINE, EMBASE and Cochrane Central to identify relevant randomized controlled trials (RCTs) investigating the effects of tacrolimus as a rinse solution in liver transplantation. Postoperative liver function, including alanine aminotransferase (ALT), aspartate aminotransferase (AST) and total bilirubin (TBIL), at postoperative day (POD) 1, 2 and 7 was extracted for pooled estimation. Forest plots were generated to calculate the differences between the groups. The I2 index statistic was used to assess heterogeneity. Publication bias was evaluated using funnel plots and Egger's test.

**Results:**

Three RCTs including 70 liver transplants were evaluated in this study. Pooled estimation revealed that rinse with tacrolimus in liver transplantation did not provide hepatic protection with respect to postoperative ALT (Test *Z* = 1.36; *P* = .175), AST (Test *Z* = 1.70; *P* = .090) or TBIL (Test *Z* = 0.69; *P* = .490). Sensitivity analysis by excluding extended donor criteria (EDC) livers showed similar results. Funnel plots and Egger's test demonstrated that there was no substantial bias.

**Conclusion:**

We may tentatively conclude that tacrolimus is ineffective for amelioration of postoperative liver function as a rinse solution in liver transplantation. Nevertheless, there is great space for future research in this area, and the potential clinical value of tacrolimus needs to be further addressed. We are expecting more evidence to support our speculations.

## Abstract 2

Supplemental Digital Content is available in the text

## 1 Introduction

Ischaemia-reperfusion injury (IRI) is widely recognized and has considerable effects on outcomes of liver transplantation, including allograft dysfunction that may introduce high morbidity.^[1,2]^ Therefore, the establishment of clinical strategies for IRI reduction is needed. Basic science had demonstrated the pivotal role of reactive oxygen species (ROS) in the development of reperfusion injury following warm or cold hepatic ischaemia in liver transplantation.^[3,4]^ ROS-mediated immunological reactions are focused on when aiming for IRI reduction. Tacrolimus is the most widely used calcineurin inhibitor for the prevention of allograft rejection.^[5]^ It may reduce IRI by altering hepatic microcirculation, and it may also promote maintenance of microcirculation in the face of the normally deleterious reperfusion by suppressing endothelial expression of the potent vasoconstrictor endothelin-1.^[6,7]^ More importantly, tacrolimus was demonstrated to effectively ameliorate IRI through the preservation of cytosolic and extracellular glutathione (GSH)^[8]^ that could be regarded as endogenous defence system against ROS.^[9,10]^ For clinical use, tacrolimus remains the mainstay of immunosuppression following liver transplantation.^[11,12]^ It was widely administered intravenously and orally following transplants, and its use strategy is constantly being refined.^[5]^

In recent years, graft rinse with immunosuppressant in transplantation has been paid increasing amounts of attention.^[13]^ It is believed that intraoperative graft rinse in transplantations may significantly ameliorate IRI.^[14,15]^ As an effective strong immunosuppressant, tacrolimus has been used for graft rinse in liver transplantation for years. Nevertheless, whether tacrolimus could be used as a regular rinse solution in liver transplantation remains uncertain because the hepatic protective effects of tacrolimus remain largely controversial. Therefore, it is necessary to perform a comprehensive meta-analysis to determine the effects of tacrolimus as a rinse solution in liver transplantation. More importantly, this study was undertaken to provide objective options for clinical decision-making and to discover new directions for clinical trials or basic science explorations.

## 2 Methods

### 2.1 Literature search and retrieval

Current meta-analysis was based entirely on previous published studies which had declared ethical approvals and no original clinical raw data were collected or utilized, thereby ethical approval was not conducted for this study. What's more, this study was initiated with strict accordance with previously established PRISMA guidelines,^[16]^ and it has been registered online in PROSPERO with ID 108191. We conducted the literature retrieval only in globally recognized databases, namely, MEDLINE, EMBASE, and Cochrane Central, to ensure the authority of the results. Relevant MeSH terms were individually or searched in combination to identify relevant studies (the search strategy is presented in Supplementary Table S1). We did not apply any restriction of publication time or status; nevertheless, the full text had to be evaluated if it was to be considered for inclusion.

### 2.2 Inclusion and exclusion criteria

We defined the following items as inclusion criteria: randomized controlled trials (RCTs); studies focusing on liver transplantation; reported available parameters of interests; equal basic treatment between the intervention group and control group.

The exclusion criteria eliminated studies with the following characteristics: non-RCTs; absence of control group or available parametric data; other graft transplantation; basic science studies; reviews, study protocols, comments, or case reports.

### 2.3 Parametric data selection and quality assessment

In the present study, we focused on the protection effects of tacrolimus in liver transplantation, thus postoperative liver function was of interest. We chose alanine aminotransferase (ALT), aspartate aminotransferase (AST), and total bilirubin (TBIL) at postoperative day (POD) 1, 2, and 7 as parametric data. Full texts of eligible RCTs were reviewed in detail to extract general information (e.g., author's name, year of publication), and parametric raw data were recorded for final quantitative analysis.

For quality assessment, included trials were assessed by the Cochrane Risk of Bias assessment tool^[17]^ to address the bias risk of individual studies according to selection, performance, detection, attrition, reporting, and other bias. A graphic summary of the overall and study-level risk of bias was conducted using Review Manager Software (Version 5.3, Cochrane Community, United Kingdom). The process of data extraction and quality assessment was performed by group discussion to reach agreement.

### 2.4 Statistical analysis

For meta-analysis, the values of ALT, AST, and TBIL at POD 1, 2, and 7 were selected for pooled estimation. In this condition, heterogeneity (*I*^2^ index statistic) in the study design was used to estimate a data mode for using fixed- (*I*^2^ < 50%) or random- (*I*^2^ > 50%) effects models.^[18]^ The associated 95% confidence intervals (CIs) were calculated, and the level of statistical significance was set at *P* < .05. Data were expressed as the mean plus standard deviation. Publication bias was assessed by examining funnel plot symmetry and performing Egger test. For studies presenting median and range values, the raw data were converted to mean plus standard deviation by Luo and Wan formulas.^[19,20]^ Values of changes would be combined with baseline for final quantitative synthesis. For trials with incomplete literal data reports, graphic data extraction would be conducted using OriginPro Graphing and Data Analysis Software (Version 9.1, OriginLab Corporation, MA).^[21]^ Data manipulation, statistical analyses of network meta-analysis, and pairwise analyses were conducted using the Stata software package (Version 12.0, StataCorp LLC, Texas).^[22]^

## 3 Results

### 3.1 Study characteristics and bias assessments

**Figure 1:**
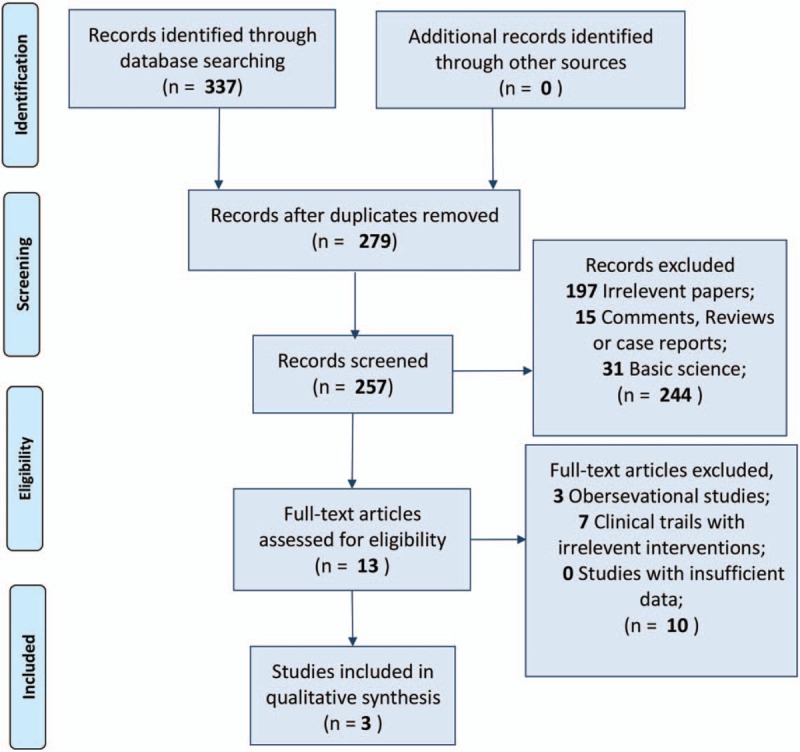
Flow diagram of the process of including and excluding studies for this meta-analysis.

After detailed review, we identified 337 relevant studies and 3 RCTs^[23–25]^ including 70 liver transplantation cases for final meta-analysis (Fig. 1). One of these 3 trials was from the USA and the other 2 were from Europe. All the tacrolimus solutions in the respective trials used the same concentration (20 ng/mL) (Table 1). For bias assessment, random sequence generation was clear in only one RCT; 2 of the 3 trials were based on a double-blinded process (details in Supplementary Figure S1).

**Table 1:**
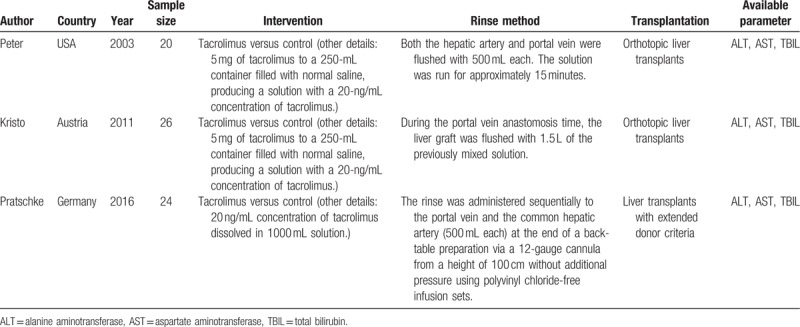
Brief description of characteristics for the included trials.

### 3.2 Tacrolimus revealed no hepatic protective effects in liver transplantation

**Figure 2:**
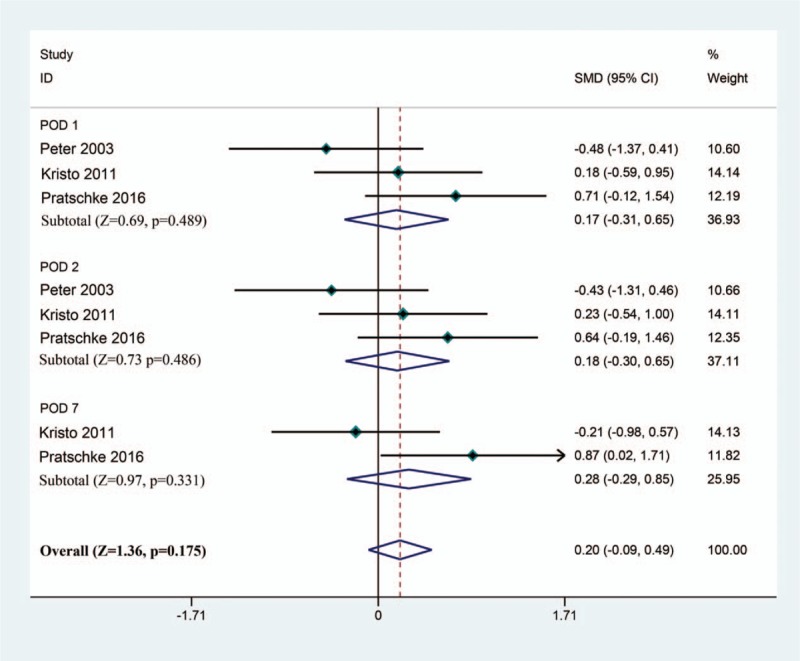
Pooled estimation of ALT levels at POD 1, 2, and 7 between the tacrolimus and control groups. ALT = alanine aminotransferase, POD = postoperative day.

**Figure 3:**
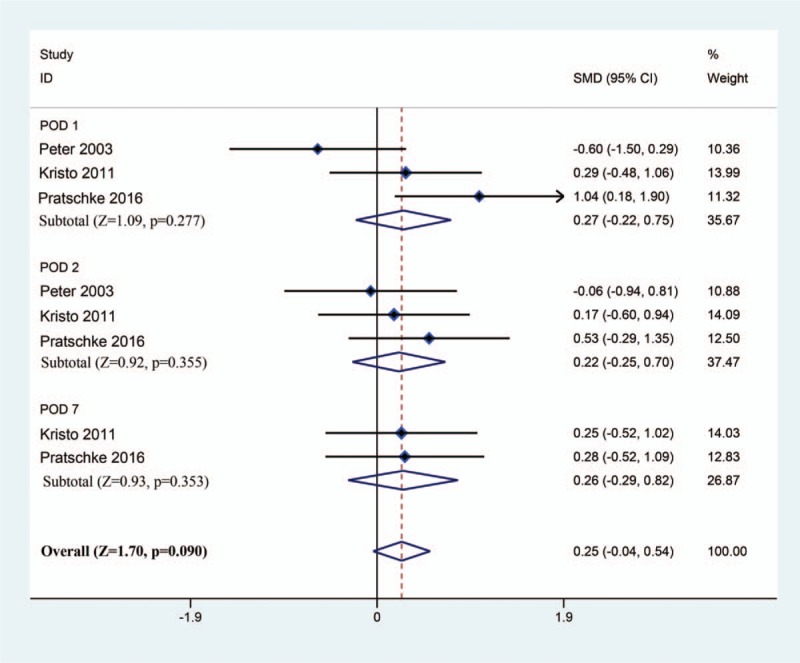
Forest plot between the tacrolimus and control groups with respect to AST levels at POD 1, 2, and 7. AST = aspartate aminotransferase, POD = postoperative day.

**Figure 4:**
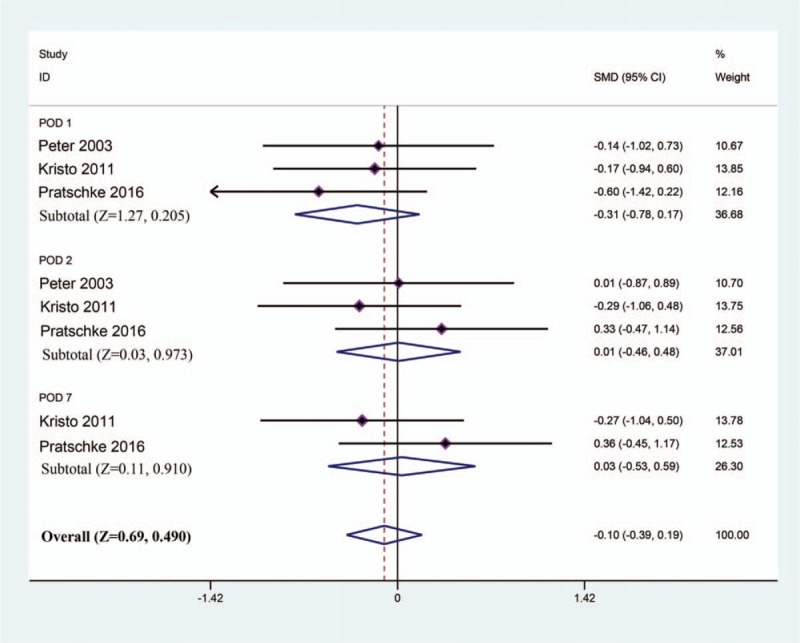
Meta-analysis of TBIL values at POD 1, 2 and 7 between the tacrolimus and control groups. POD = postoperative day, TBIL = total bilirubin.

To investigate the hepatic protective effect of tacrolimus as a rinse solution in liver transplantation, we compared the liver function (including ALT, AST, and TBIL) at POD 1, 2, and 7. For postoperative ALT, based on a fixed model (*I*^2^ = 31%), the results of meta-analysis revealed no significant difference between the tacrolimus group and the control group after liver transplantation (SMD [95% CI] = 0.20 [–0.09, 0.49]; Test *Z* = 1.36; *P* = .175) (Fig. 2). For postoperative AST, all 3 RCTs reported relevant data. After pooled estimation, we discovered that there was no significant difference between the tacrolimus and control groups at postoperative day 1, 2, and 7 (SMD [95% CI] = 0.25 [–0.04, 0.54]; Test *Z* = 1.70; *P* = .090) based on a fixed model (*I*^2^ = 9%) (Fig. 3). Finally, we comprehensively evaluated the TBIL at postoperative day 1, 2, and 7 based on a fixed model (*I*^2^ = 0%). We found that there was no marked impact on postoperative TBIL using tacrolimus as a rinse solution in liver transplantation (SMD [95% CI] = –0.10 [–0.39, 0.19]; Test *Z* = 0.69; *P* = .490) (Fig. 4). In summary, the results of meta-analysis implicated that there was no difference between the tacrolimus and control groups with respect to postoperative ALT, AST, and TBIL. This may indicate that tacrolimus rinse in transplantation exhibited limited hepatic protective effects.

### 3.3 Sensitivity analysis

Despite using the same concentration of tacrolimus as a rinse solution in liver transplantation, we noticed that one of the included RCTs was based on extended donor criteria (EDC)^[25]^ while the other 2 were based on regular orthotopic liver transplants (Table 1). To accurately elucidate the impact of tacrolimus rinse on hepatic function, we performed sensitivity analysis regarding same parametric data by excluding the trial of EDC transplants. We found that tacrolimus exhibited no hepatic protective effect as a rinse solution in liver transplantation with respect to postoperative ALT (SMD [95% CI] = –0.10 [–0.47, 0.26]; Test *Z* = 0.57; *P* = .572) (Supplementary Figure S2), AST (SMD [95% CI] = 0.05 [–0.32, 0.41]; Test *Z* = 0.26; *P* = .796) (Supplementary Figure S3) or TBIL (SMD [95% CI] = –0.18 [–0.55, 0.18]; Test *Z* = 1.00; *P* = .319) (Supplementary Figure S4).

### 3.4 Publication bias

As mentioned above, the 3 RCTs did not reveal substantial risk of bias. To further explore potential publication bias, we conducted quantitative calculations based on funnel plots; we also performed Egger test to detect potential publication bias. We then observed funnel plot asymmetries with respect to ALT, AST, and TBIL. The statistical outcomes of Egger tests showed no bias (Supplementary Figure S5–7).

## 4 Discussion

To perform our meta-analysis, we conducted retrieval from global authoritative databases and found 3 RCTs containing 70 liver transplantation cases for final quantitative analysis. After comparing ALT, AST, and TBIL at postoperative day 1, 2, and 7, we discovered no significant difference between the tacrolimus and control groups, suggesting that intraoperative graft rinse with tacrolimus may not ameliorate postoperative liver function. Furthermore, sensitivity analysis revealed similar results after excluding the trial based on EDC livers. In addition, funnel plots and Egger tests regarding each parameter demonstrated no marked bias. In terms of the objective results, we may tentatively conclude that intraoperative rinse with tacrolimus may not be useful in liver transplantation. Nevertheless, some deeper facts need to be further discussed.

Investigations with cultured hepatocytes from human samples have documented the membrane-stabilizing effects of intravenous tacrolimus leading to tacrolimus being a widely used immunosuppressant in liver transplantation.^[26]^ Its preclinical theoretical basis was that animal models had shown decreased cellular injury from ischaemia and reperfusion after treatment with tacrolimus.^[6,27,28]^ This was the reason that tacrolimus was considered as an intraoperative rinse solution. Nevertheless, it appeared that intraoperative graft flushing with tacrolimus provided no hepatic protective effects in liver transplantation. As mentioned above, tacrolimus is a calcineurin inhibitor with potential benefit in terms of attenuating IRI.^[8–10]^ Nevertheless, tacrolimus may also bring risk of hypertension, nephrotoxicity, and possibly myocardial ischaemia through its potential vasoconstrictive activity.^[29,30]^ It could even lead to cholestasis and hepatotoxicity after liver transplantation.^[31]^ Most of these risks are due to its molecular interactions at inappropriate doses.^[32]^ For tacrolimus, various dispersion concentrations in donor liver tissue may bring different consequences; therefore, the crucial factor of tacrolimus therapy may be the balance between immunosuppression and toxic overdose.^[33]^ As an intraoperative rinse solution, intraoperative flushing may provide an immediate-release of extracellular dose that was fixed in all reported RCTs (20 ng/mL). Various graft qualities, patient characteristics, and sensitivities to tacrolimus were also involved. These parallel factors may contribute to the imbalance of the abovementioned immunosuppression and overdose-induced toxicity. This may be one of the reasons that intraoperative tacrolimus rinse could not provide any benefits. On the other hand, no uniform standard clinical flush technique was used, even though the graft was rinsed with same concentration of tacrolimus. It had been determined that various flushing techniques may provide varying postoperative outcomes for liver transplantation.^[34,35]^ Prior to liver transplant, various rinse techniques may cause differences in the amount and concentration of intrahepatic tacrolimus. These discrepancies may lead to variable sensitivities to IRI. Furthermore, other details such as temperature control, residual preservation solution, or liver source may have disparate impacts on the hepatic protective effects of tacrolimus rinse solutions. Therefore, we recognize that many factors may explain our results.

For the first time, we conducted a quantitative analysis of postoperative liver function to evaluate the hepatic protective effects of tacrolimus as a rinse solution in liver transplantation. In summary, it appeared that tacrolimus rinse in liver transplantation failed to ameliorate ischaemia-reperfusion injury; there was no protection of postoperative liver functions regarding ALT, AST, and TBIL. Meanwhile, some other parametric data, such as international normalized ratio (INR) and partial thromboplastin time (PTT), also implied that tacrolimus was unable to reveal protects against IRI.^[23,24]^ But additional pooled estimation could not be conducted due to inadequate raw data. This result appears to contradict those of previous clinical trials and animal experiments. Nevertheless, based on the abovementioned factors, we may raise some hypotheses and directions for future investigations. First, as we described previously, all included RCTs were performed with the same concentration of tacrolimus. Furthermore, uniform standards for rinse technique are inadequate, leading to disorderly flush sequences and vessel paths. In addition, other details including rinse time, rinse solution amount, and precise temperature control appeared to have no corresponding attention. Therefore, we should develop a complete set of tacrolimus rinse programme standards in the future, and the effects of the programme may bring different clinical benefits. Second, tacrolimus may cause extra injury to EDC livers.^[27]^ We may doubt whether tacrolimus remains worth using for EDC livers; in addition, whether readjustment of the rinse programme will alter this condition remains uncertain. Furthermore, tacrolimus was proved to suggested double-edged effects. The development of new agents combined with tacrolimus treatment in liver transplantation as a novel mixed rinse solution to eliminate its negative effects appears to be another research area. Finally, uncertain clinical effects of tacrolimus may urge the development of relevant basic science research to perfect the theoretical basis for tacrolimus. All these deep discussions may introduce new ideas and directions for future studies; nevertheless, we admit some shortcomings requiring elaboration. There were only 3 RCTs containing 70 cases for analysis, possibly making our conclusion unstable. Furthermore, many confounding factors may interfere with our conclusions despite that fact that we conducted deep discussions. Finally, despite negative results from Egger test, we believed there were some potential biases in this study, and the conclusions need to be further clarified. We are expecting more RCTs in the future.

In general, based on the current objective results, we temporarily conclude that tacrolimus as a rinse solution is ineffective for alleviation of IRI in liver transplantation. Nevertheless, we conducted deep analysis according to the results and believe that there is great space for future research in this area. The potential clinical value of tacrolimus needs to be further addressed. We are also expecting further evidence to support our conclusions.

## Acknowledgments

The authors report no financial relationships with commercial interests.

## Author contributions

**Conceptualization:** Tao Guo.

**Data curation:** Tao Guo, Junhao Lei, Jiamin Gao.

**Formal analysis:** Tao Guo.

**Investigation:** Tao Guo, Junhao Lei, Jiamin Gao.

**Methodology:** Tao Guo, Junhao Lei.

**Software:** Tao Guo, Jiamin Gao.

**Supervision:** Zhen Li, Zhisu Liu.

**Validation:** Tao Guo, Zhen Li.

**Writing – original draft:** Tao Guo.

**Writing – review & editing:** Tao Guo, Zhen Li, Zhisu Liu.

## Supplementary Material

Supplemental Digital Content
