# Supplementary material for: The hepatic protective effects of tacrolimus as a rinse solution in liver transplantation: A meta-analysis
Source: Medicine (Baltimore). 2019 May 24;98(21):e15809. doi: 10.1097/MD.0000000000015809 (PMC6571202; doi:10.1097/MD.0000000000015809)

Supplementary Table and Figures

Table S1. Search strategy in MEDLINE.

No.	Search	Results
1	Exp (tacrolimus) OR FK506/	24111
2	Exp (((flush) OR infusion) OR reperfusion) OR rinse /	317426
3	Exp ischemia/	267323
4	1 AND 2 AND 3	259
5	Exp ((liver) OR hepatic) OR graft/	1387863
6	Exp transplantation	704949
7	5 AND 6	281059
8	Exp ((prospective) OR random) OR randomized controlled trial/	1618893
9	4 AND 8	231
10	7 AND 8	355
12	4 AND 7 AND 8	107

Figure S1. Bias assessment for included trials. (A) Risk of bias graph presented as percentages across all included studies; (B) Judgments regarding each risk of bias item for each included study.

A

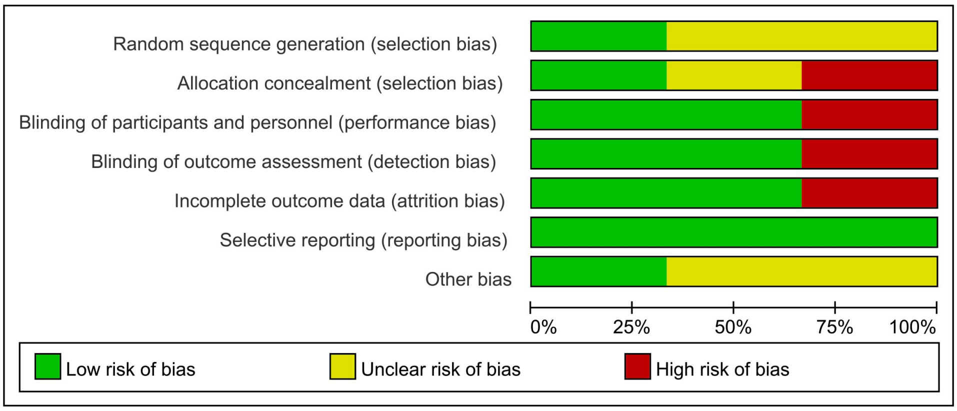

B

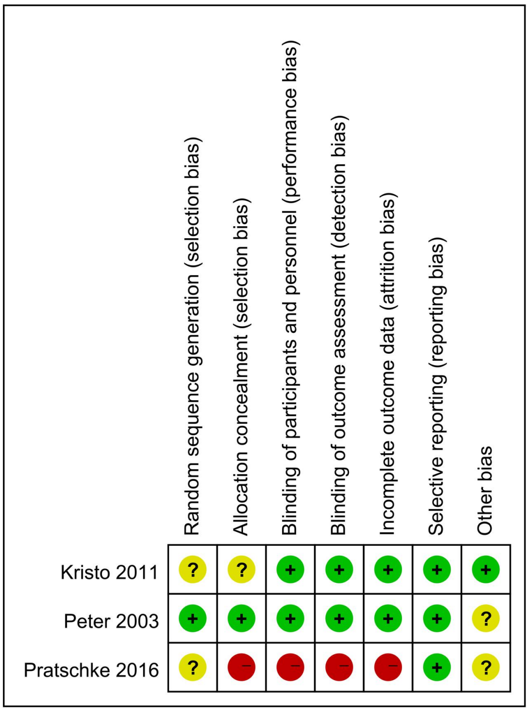

Figure S2. Sensitive analysis regarding ALT. [POD: postoperative day.]

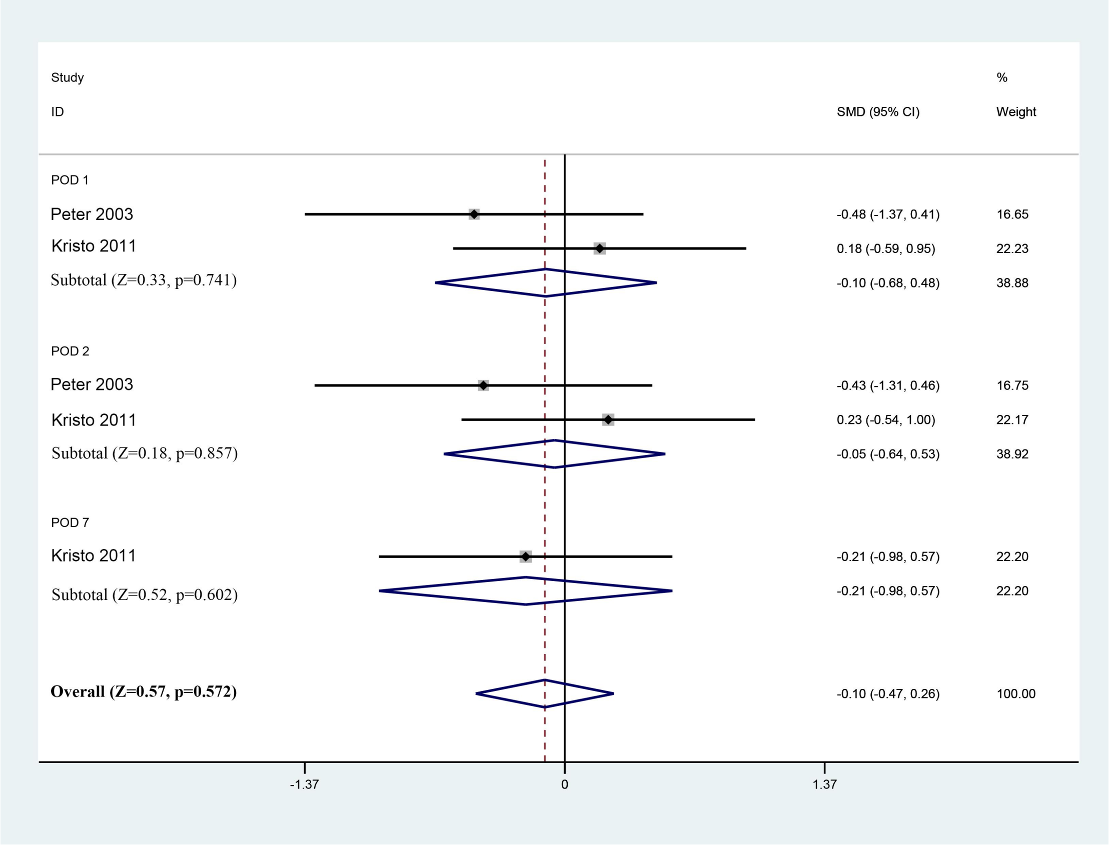

Figure S3. Sensitive analysis regarding AST [POD: postoperative day.]

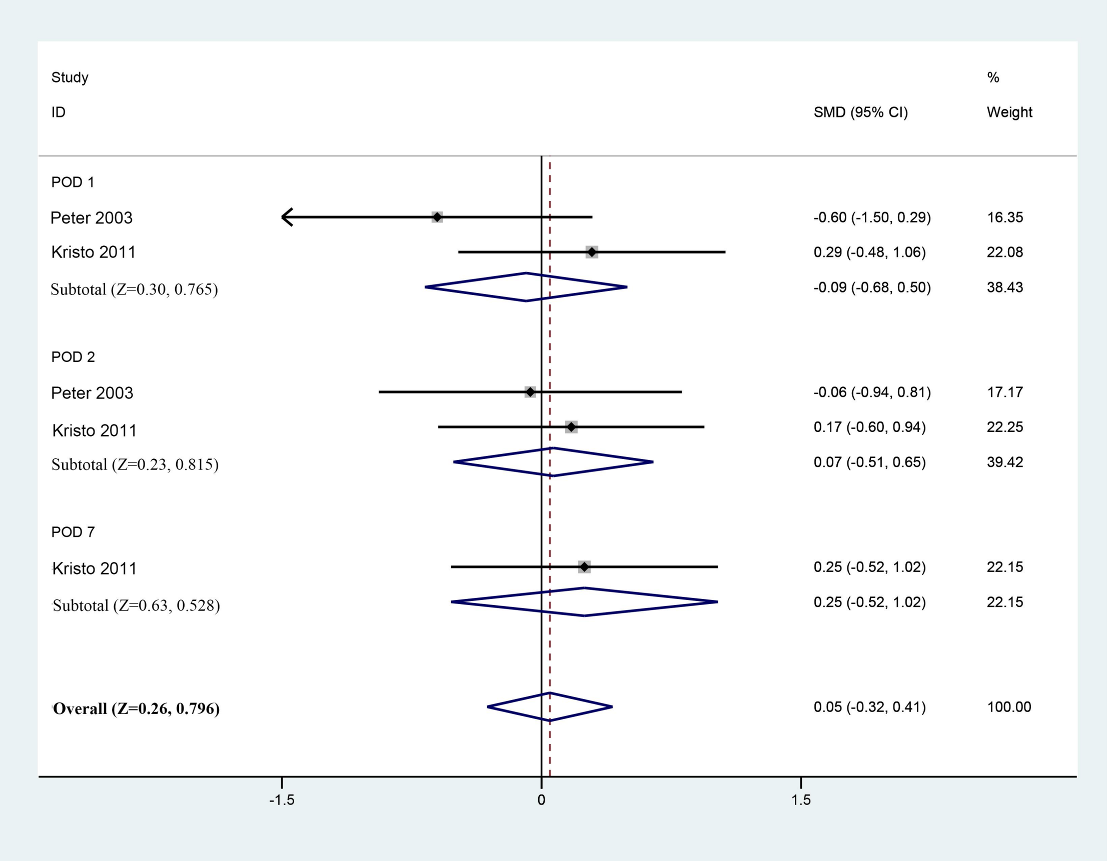

Figure S4. Sensitive analysis regarding TBIL. [POD: postoperative day.]

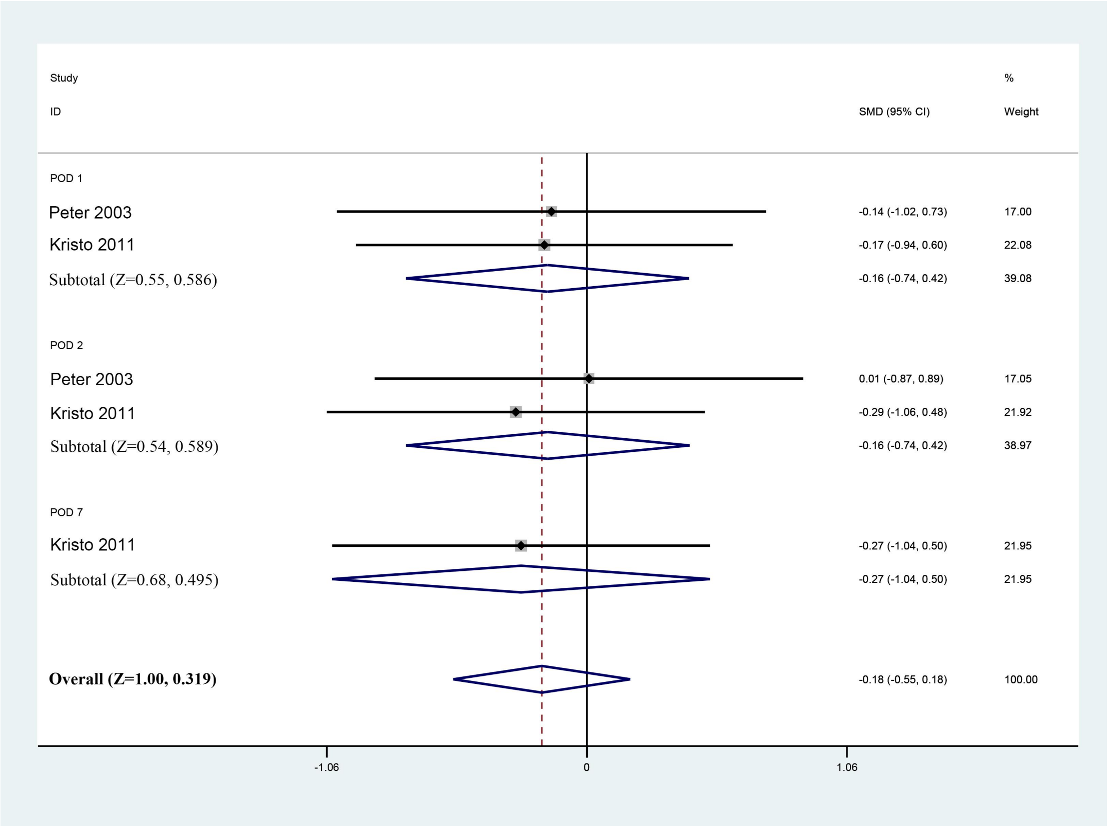

Figure S5. Bias evaluation based on (A) funnel plot, and (B) Egger's publication bias plot regarding ALT.

A

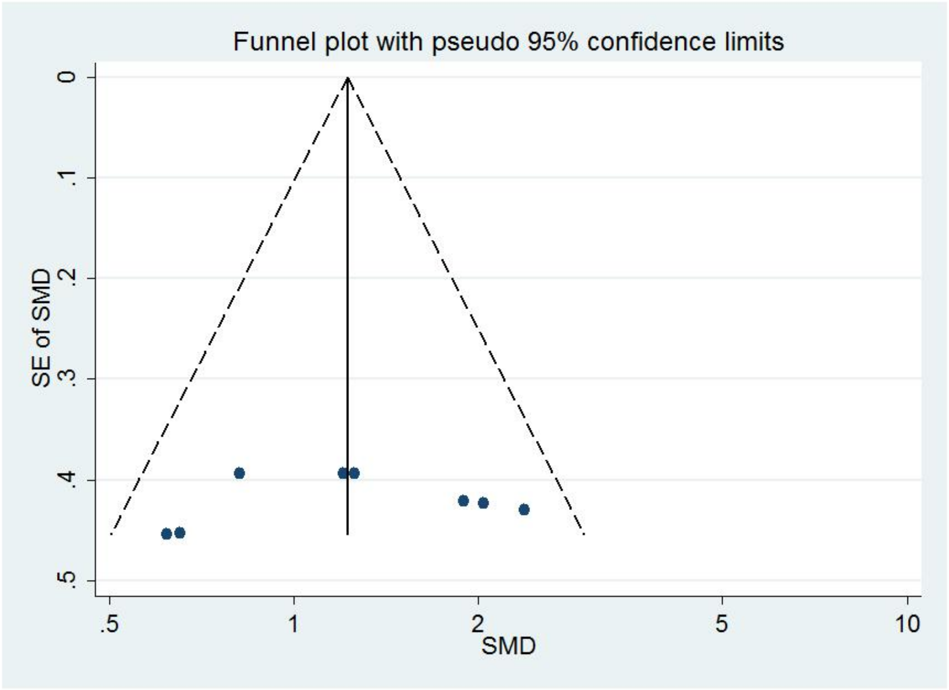

B

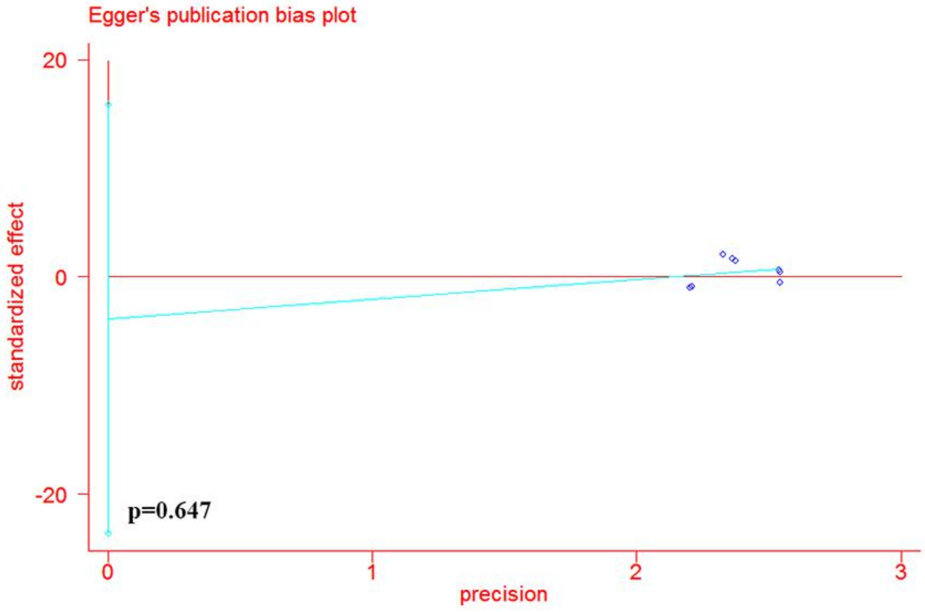

Figure S6. Bias evaluation based on (A) funnel plot, and (B) Egger's publication bias plot regarding AST.

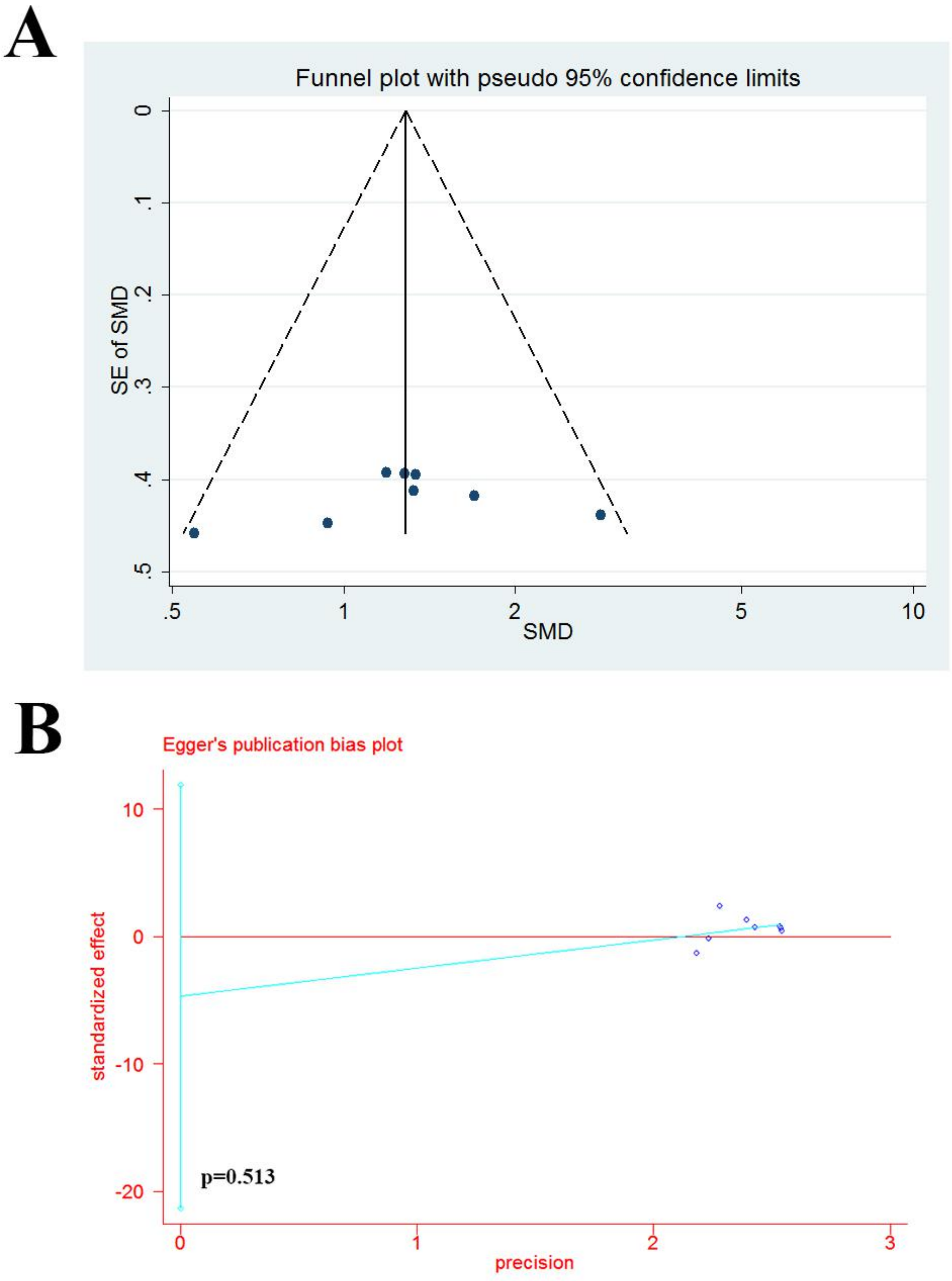

Figure S7. Bias evaluation based on (A) funnel plot, and (B) Egger's publication bias plot regarding TBIL.

A

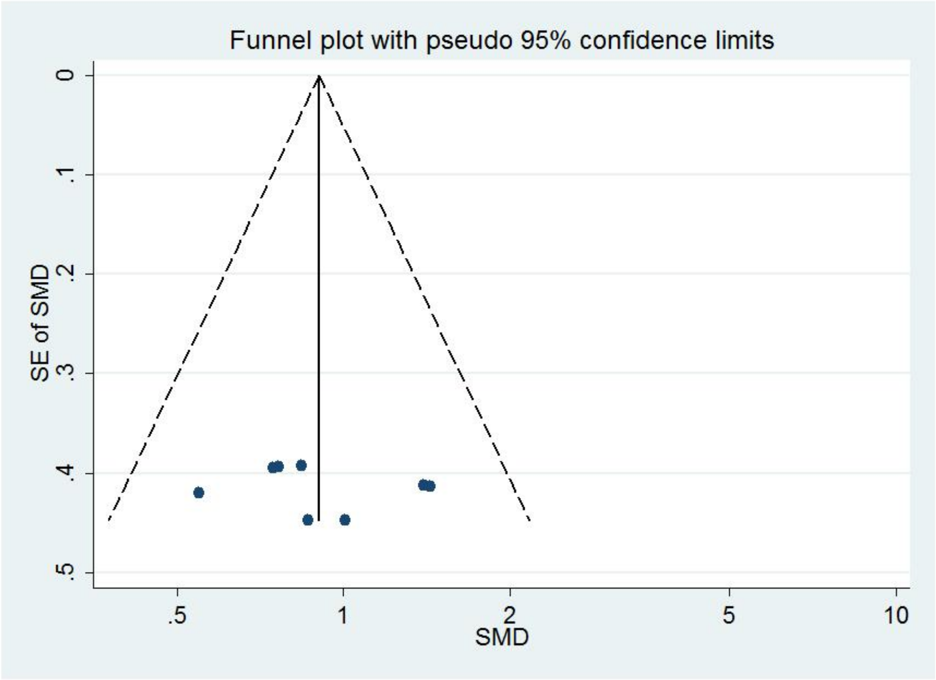

B

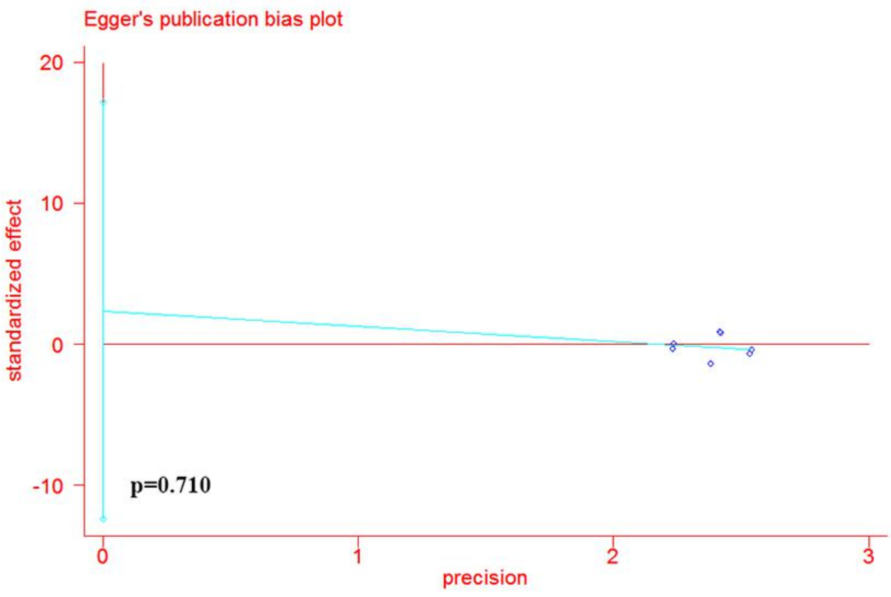

Supplement: Supplemental Digital Content [file medi-98-e15809-s001.pdf]
